# Journey of Marijuana From “Folk Tales” to “Doors of Justice”: A Comprehensive Review

**DOI:** 10.7759/cureus.57190

**Published:** 2024-03-29

**Authors:** Apurva Bezalwar, Pradeep S Patil, Shobha Pajai, Anshita Girdhar

**Affiliations:** 1 Psychiatry, Jawaharlal Nehru Medical College, Datta Meghe Institute of Higher Education and Research, Wardha, IND; 2 Physiology, Mahatma Gandhi Institute of Medical Sciences, Wardha, IND

**Keywords:** social equity, public health, regulation, legalization, india, marijuana

## Abstract

This comprehensive review delves into the intricate journey of marijuana in India, tracing its historical and cultural significance from ancient times to the present day. Despite its deep-rooted presence in religious rituals, traditional medicine, and cultural festivities, marijuana faces stringent prohibition under the Narcotic Drugs and Psychotropic Substances Act 1985. This review critically examines the current legal framework, highlighting its societal impacts and limitations. Through an evidence-based analysis, it advocates for a reevaluation of marijuana laws to align with contemporary realities, promoting public health, social equity, and economic development. By envisioning a future of evidence-based regulation and innovation, India can unlock the full potential of marijuana as a “miracle crop” for the betterment of its people and society.

## Introduction and background

Marijuana, known as "Ganja" or "Bhang" in India, holds deep historical and cultural roots within the country. Dating back thousands of years, marijuana has been integral to various religious, spiritual, and medicinal practices. In ancient texts, such as the Vedas and the Atharvaveda, references to cannabis as a sacred herb are abundant, depicting its use in rituals, ceremonies, and Ayurvedic medicine [1]. Throughout Indian history, marijuana has been revered for its purported medicinal properties, believed to alleviate ailments ranging from pain and inflammation to anxiety and insomnia. Additionally, it has played a significant role in cultural festivities, particularly during festivals like Holi and Mahashivratri, where bhang-infused beverages are customary [2].

Despite its deep cultural significance, the legal status of marijuana in India is largely prohibitive. The Narcotic Drugs and Psychotropic Substances (NDPS) Act 1985 categorizes cannabis as a Schedule I drug alongside substances like heroin and cocaine, indicating a high potential for abuse and strict legal penalties for possession, cultivation, and trafficking [3]. Under the NDPS Act, the possession, sale, transportation, and cultivation of marijuana are criminal offenses, punishable by imprisonment and fines. However, certain exceptions exist for the use of cannabis leaves and seeds for industrial or horticultural purposes, subject to stringent regulations [4].

The purpose of this comprehensive review is to critically examine the legal, social, economic, and health implications of marijuana prohibition in India. By delving into the historical context, current legal framework, and cultural significance of marijuana, this review seeks to assess whether existing laws align with contemporary realities and societal needs. Furthermore, this review aims to explore the potential benefits of legalizing and regulating marijuana, considering its medicinal properties, economic potential, and impact on public health. An evidence-based analysis intends to provide insights and recommendations for policymakers, legislators, and stakeholders to reconsider and reformulate marijuana policies in India.

## Review

Historical background

Traditional and Cultural Uses of Marijuana in Ancient India

In ancient India, cannabis, referred to as "bhang," held various traditional and cultural roles deeply ingrained in Hindu customs and traditional medicine. The plant was utilized for spiritual and medicinal purposes for thousands of years. References to cannabis in ancient Hindu scriptures like the Vedas underscore its importance as one of the five sacred crops, alongside barley and soma, with mentions dating back to 1500-500 BC [5]. Cannabis found its place in Hindu legends, particularly those revolving around the deity Shiva, which is often associated with the consumption of bhang. Narratives depict instances where cannabis played a role in religious rituals or served as a remedy for ailments. For instance, in Hindu mythology, when Shiva ingested poison during the “Samudra Manthan,” bhang was administered to alleviate his suffering [6]. Medicinally, cannabis was acknowledged for its therapeutic properties in ancient India. The earliest references to bhang as a medicinal substance can be traced back to the works of Sushruta around 500-600 AD, where it was described as "anti-phlegmatic" and recommended for various conditions such as catarrh and biliary fever. Ayurveda, the traditional Indian system of medicine, utilized cannabis primarily for issues related to the digestive and respiratory tracts [7]. In addition to its medicinal and spiritual uses, ancient Indians also utilized the hemp plant for industrial purposes. Hemp fibers were employed in textile production, including clothing, and evidence suggests that ancient Indian artisans may have been early practitioners of hempcrete technology [8]. The historical context of cannabis in India paints a multifaceted picture of religious, medicinal, and industrial significance that has persisted over millennia despite changes in legal frameworks and societal attitudes toward the plant [9].

Influence of Religious and Spiritual Practices on Marijuana Consumption

Religious and spiritual practices exert a significant influence on marijuana consumption, showcasing diverse perspectives and impacts across various belief systems. Within many religious traditions, cannabis has been seamlessly integrated into rituals and ceremonies as a tool for spiritual exploration, personal transformation, and forging connections with the divine. For instance, in Rastafarianism, cannabis assumes a central role as a sacrament, symbolizing spiritual enlightenment and cultural identity [10]. Similarly, contemporary religious movements, such as neo-paganism, the Native American Church, Santo Daime, and certain branches of Buddhism, have embraced cannabis in their practices to augment meditation, foster reverence for nature, and facilitate spiritual encounters [10]. The research underscores the notion that religious involvement can shape attitudes toward marijuana use. Studies suggest that heightened religious engagement correlates with lower rates of marijuana use, particularly for recreational purposes [11]. However, the impact of religious beliefs on marijuana consumption can fluctuate depending on individual health status and usage context. While some religious teachings may discourage substance use, including marijuana, others regard cannabis as a natural substance capable of facilitating higher states of consciousness [11]. In essence, the relationship between religion, spirituality, and marijuana consumption is intricate and multilayered. While some individuals perceive cannabis as a means to deepen their spiritual experiences or diverge from conventional religious doctrines, others view it as incongruent with their spiritual values or detrimental to their well-being [10-12]. The interplay between religious beliefs and marijuana use underscores the necessity for further research to comprehend how various faith traditions mold attitudes toward cannabis in diverse cultural milieus.

Colonial Era and the Introduction of Restrictive Laws

During the colonial era, the British rulers of India initiated the large-scale cultivation of cannabis for hemp, leading to a rapid expansion of cannabis cultivation and utilization in India and worldwide [13]. This surge in cannabis use eventually prompted the creation of the Indian Hemp Drugs Commission report in 1894, one of the earliest systematic studies on cannabis use. The report concluded that only heavy use had significant adverse effects [13]. However, despite its historical significance and widespread use, India enacted the NDPS Act in 1985, effectively prohibiting the cultivation, production, and consumption of cannabis alongside other narcotic and psychotropic drugs [7]. The consumption of cannabis derivatives, particularly bhang, carries significant religious and cultural importance among Indians and remains widely accepted in Indian communities [7]. Despite legal restrictions, cannabis continues to be one of the most consumed and trafficked illicit substances in India, with cities like Mumbai and Delhi ranking among the top ten globally for cannabis consumption [7]. The momentum for legalizing cannabis in India for both medical and recreational purposes has been steadily growing, with efforts directed toward its medicinal and commercial utilization in certain states such as Uttarakhand and Madhya Pradesh [7].

Current legal framework

Overview of the NDPS Act 1985 Background of Opium in the Indian Context

Previously, matters about drugs were addressed through a series of legislative acts. The Opium Act of 1857 and the Opium Act of 1878 exclusively targeted opium, while the Dangerous Drugs Act of 1930 addressed cannabis and cocaine. The Drugs and Cosmetics Act of 1940 partially covered other drugs, with no mention of cannabis cultivation, and the maximum punishment was three years with or without a fine [14]. India lacked legislation specifically targeting narcotics until 1985. The “Indian Hemp Drugs Commission 1893” deemed moderate cannabis use acceptable, and cannabis remained legally available for sale until 1985. However, following the United Nations (UN) adoption of drug laws in 1961, the United States advocated for global prohibition against all drugs. Despite India's resistance for nearly 25 years, it eventually succumbed to pressure and accepted the UN Convention on Drugs Act in 1985. Consequently, the NDPS Act was enacted by the Indian Parliament in 1985. The responsibility for controlling drug abuse falls under the Ministry of Finance and the Department of Revenue [14].

The NDPS Act, enacted on November 14th, 1985, serves to regulate and control narcotic drugs and psychotropic substances in India [14,15]. The act, numbered 61 of 1985, has undergone several amendments in 1989, 2001, 2011, 2014, and 2015. This legislation prohibits the manufacture, cultivation, possession, sale, purchase, transport, storage, or consumption of drugs without permission from the appropriate authorities [14]. Consisting of 83 sections divided into six chapters or parts, the NDPS Act covers various aspects, including definitions, authorities and officers, prohibition, control, regulation, offenses, penalties, and procedures for search and seizure [14].

Objectives of the act include amending and consolidating laws related to narcotic drugs, establishing stringent provisions for control and regulation of illegal drug activities, providing mechanisms for forfeiting illicit substances and properties used in drug trafficking, and implementing international conventions on narcotic drugs [14]. The severity of punishment under the NDPS Act is determined by the quantity of seized drugs, categorized into small quantities, more than small but less than commercial quantities, and commercial quantities defined within the act [16]. The act aims to strengthen controls over drug abuse, enhance penalties for trafficking offenses, and align with international conventions on narcotic drugs and psychotropic substances, to which India is a party [16]. Additionally, provisions are made for the establishment of centers for the identification and treatment of people with an addiction, as well as the supply of narcotic drugs and psychotropic substances [17].

Classifications of Drugs Under the NDPS Act and Implications for Marijuana

Under the NDPS Act in India, cannabis is categorized into different classifications. Cannabis, commonly referred to as hemp, encompasses Charas (resin obtained from the cannabis plant), Ganja (flowering or fruiting tops of the cannabis plant), and any mixture or drink prepared from these forms [18]. The NDPS Act strictly prohibits the production, possession, sale, purchase, transport, storage, and consumption of narcotic drugs such as marijuana, hashish, and bhang in India [18]. However, there are exceptions within the act; for example, bhang is exempted due to its cultural significance in Hinduism and its association with festivals like Holi [18]. The act permits the cultivation of cannabis plants for industrial purposes, such as obtaining fiber or seeds, under specific regulations set by the government [18]. Punishments under the NDPS Act vary depending on the quantity of the prohibited substance involved. For small quantities, imprisonment of up to six months or a fine of up to Rs. 10,000 may be imposed; for quantities less than commercial but more than small, imprisonment can extend to 10 years with a fine of up to Rs. 1 lakh; and for commercial quantities, imprisonment can range from 10 to 20 years with a fine of up to Rs. 2 lakhs [19]. Despite ongoing debates surrounding the legalization of cannabis in India for various purposes, the current legal framework remains stringent regarding its production and use [20].

Legal Status of Medical and Recreational Marijuana

The legal landscape surrounding medical and recreational marijuana in India is complex. While the cultivation of cannabis for industrial purposes, such as producing industrial hemp, is permitted, the recreational use of cannabis is effectively prohibited under the NDPS Act of 1985 [20,21]. Research into the medical applications of cannabis received legalization approval only in 2017, representing a significant milestone in exploring its therapeutic potential [21,22]. Despite increasing interest and discussions regarding the legalization of recreational cannabis use, experts caution that India may not be adequately prepared for such a shift due to challenges within the criminal justice and healthcare systems [21,23]. Current laws tightly regulate the possession, sale, and production of cannabis products, with individual states having their regulations and amendments concerning cannabis use [20,22]. The debate over whether to relax regulations for medical and industrial cannabis uses or to permit recreational use continues, with considerations revolving around potential medicinal and economic benefits weighed against concerns about substance abuse [20,23]. Overall, while there exists a burgeoning industry for hemp products and CBD in India, the legalization of recreational cannabis remains a contentious issue that demands a thorough examination of its various implications [22].

Social and economic impact

Analysis of the Impact of Marijuana Prohibition on Society

Legalizing cannabis offers several potential benefits and considerations that warrant careful examination. Firstly, it can significantly save law enforcement costs associated with enforcing prohibition laws. For example, California saw a substantial 74% reduction in yearly spending on enforcing marijuana laws after repealing prohibition of use [24]. However, the prohibition of marijuana has led to the criminalization of millions of African Americans, with enforcement disproportionately impacting specific demographics. Studies reveal that African Americans are over three times more likely to be arrested for marijuana-related offenses despite similar usage rates as whites [24]. Furthermore, the underground marijuana market poses health risks due to the lack of quality control standards, potentially resulting in contamination with harmful substances like pesticides or molds. Criminalization may also hinder harm reduction measures, such as using vaporizers to reduce respiratory risks [24]. Advocates of legalization argue for its economic benefits and potential tax revenue generation. They assert that legalization can reduce crime, lower criminal justice expenditures, improve public health, enhance traffic safety, and stimulate the economy. However, critics express concerns about potential increases in drug use and crime rates, diminished traffic safety, and negative impacts on public health [25]. Moreover, legalization may have implications for public health, including mental health, physical health, and social well-being. Therefore, it is crucial to consider the potential effects on mental health, substance abuse rates, and overall societal well-being when evaluating the legalization of recreational cannabis [21].

The Economic Potential of Legalizing Marijuana Cultivation and Trade

The legalization of marijuana cultivation and trade in India presents significant economic opportunities. With its favorable climatic conditions and extensive history of cultivation, India could competitively produce medical-grade marijuana, making it appealing in the international market [26]. The global medical cannabis market is forecasted to reach $73.6 billion by 2027, offering substantial revenue prospects for India through exports and domestic industry expansion [26]. Additionally, legalizing and regulating medical marijuana could diminish the illicit drug trade, potentially disrupting illegal networks and bolstering national security [26]. Furthermore, legalizing cannabis could spur job creation, generate additional tax revenues, and foster economic growth within the domestic industry [26]. India's well-established pharmaceutical sector could capitalize on its expertise to become a global leader in producing standardized and effective cannabis-based medicines [26]. By tapping into the burgeoning global medical cannabis market, India could bolster its economy and contribute to advancements in the phytomedicine sector [26]. Nevertheless, concerns persist regarding potential risks associated with legalization, such as increased drug abuse among adolescents and youth, reinforcement of illegal producer-supplier networks, and challenges in controlling the quality and marketing of cannabis products [27]. Addressing these risks would necessitate stringent regulations on the cultivation, production, sale, quality control, and marketing of cannabis products [21]. Public health initiatives would also be critical to educate the public about safe cannabis use and mitigate potential health risks linked with legalization [21].

Comparison With Countries That Have Legalized Marijuana

Several countries, including Canada, Uruguay, and Malta, have taken the bold step to legalize recreational cannabis at the national level. In contrast, others, such as Georgia, Germany, Luxembourg, Mexico, South Africa, Thailand, and the United States, have also moved toward legalization to varying extents [28]. The impact of marijuana legalization in different countries has been a topic of heated debate, with concerns raised about potential repercussions such as lower educational achievement among teens [25]. Research suggests that the effects of state-level marijuana legalization are not as drastic as advocates or critics often claim. While proponents of legalization emphasize benefits such as reduced crime rates and increased tax revenue, critics caution against potential negative consequences like heightened drug use and crime rates. However, the actual impact of legalization has generally been more moderate than initially anticipated [25]. The available data offer valuable insights into what other states or countries might expect from marijuana legalization or related policies. By examining the experiences of jurisdictions that have already legalized cannabis, policymakers can better understand the potential benefits and challenges associated with such measures, thereby making more informed decisions about marijuana policies in their respective regions.

Health considerations

Medical Benefits of Marijuana and Its Potential Applications

Medical benefits: Cannabis, particularly compounds like cannabidiol (CBD) and delta-9-tetrahydrocannabinol (THC), has demonstrated significant potential in managing various medical conditions. Studies have shown promise in using cannabis for chronic pain management, alleviating symptoms of nausea, managing multiple sclerosis symptoms, and addressing mental health conditions such as anxiety [29,30]. Additionally, research indicates its effectiveness in treating epilepsy and reducing seizures, as well as aiding in cancer treatment by mitigating chemotherapy-induced symptoms like nausea and vomiting [29,30]. CBD, in particular, has been found to have beneficial effects on blood pressure, inflammation reduction, and preventing relapse into drug and alcohol addiction [31]. It has also been utilized as an appetite stimulant in patients suffering from conditions such as HIV, cancer, and anorexia nervosa, where nutritional deficits are common and high nutritional requirements need to be met. Cannabis, through its agonist action on CB_1_ receptors, induces increased food cravings and enjoyment. It serves as an effective analgesic for chronic pain conditions resistant to drugs like NSAIDs and opioids, including rheumatoid arthritis, peripheral neuropathy, and fibromyalgia. Rare forms of epilepsy, such as Lennox-Gastaut syndrome and Dravet syndrome, which are often unresponsive to standard anti-epileptic medications, have shown efficacy with *Cannabis sativa* in reducing seizure frequency by nearly 50%. Some studies have also demonstrated complete remission of Crohn's disease due to its anti-inflammatory properties. Cannabis has also shown effectiveness in reducing intraocular pressure, making it potentially useful in managing glaucoma. Its mechanism of action involves the suppression of proliferative cell signaling pathways, inhibition of angiogenesis, cell migration, stimulation of apoptosis, and induction of autophagy. Additionally, cannabis has shown promise in the treatment of certain cancers, particularly glioblastoma multiforme and pancreatic cancers. In psychiatric disorders, cannabis is beneficial as an appetite stimulant in patients with anorexia nervosa, showing superior response compared to amitriptyline in treating insomnia and significantly improving tic severity in childhood-onset Tourette syndrome [31].

Health risks: Despite its medicinal benefits, cannabis use is not without risks. Smoking cannabis can pose harm to the respiratory system, causing damage to blood vessels in the lungs and increasing the risk of bronchitis [29]. Additionally, cannabis use has been associated with mental health issues such as anxiety, depression, and the potential exacerbation of conditions like bipolar disorder [29]. Excessive or improper use of cannabis products can also have adverse effects on mental health and worsen certain health conditions [29].

Legal status: The legal status of cannabis significantly impacts its medical use. In the United States, cannabis is classified as a Schedule I controlled substance by the Drug Enforcement Administration, creating obstacles for researchers studying its potential health benefits [29]. Despite this classification, there is a growing interest in investigating the therapeutic potential of cannabis for various health conditions.

Risks Associated With Marijuana Use and Abuse

The risks associated with marijuana use and abuse encompass a range of negative consequences. Individuals who operate vehicles under the influence of marijuana may experience hazardous effects, including slower reactions, diminished coordination, and difficulty in vehicle operation [32]. Chronic use of cannabis can result in mental health issues such as anxiety, depression, and an elevated risk of cannabis dependence or addiction [7,33,34]. Moreover, frequent consumption of THC over an extended period can impair crucial cognitive functions such as learning and memory [34]. Prolonged cannabis use, particularly when undertaken daily or nearly daily for several months or years, can have adverse impacts on both physical and mental health. It may impair lung function, particularly if smoked, and heighten the risk of cannabis dependence or addiction [34]. Additionally, the use of products containing high levels of THC can worsen conditions associated with anxiety and depression [34]. It is imperative to acknowledge these risks and consider them when using cannabis products, enabling individuals to make informed decisions about their consumption.

Regulation and Public Health Strategies

**Figure 1:**
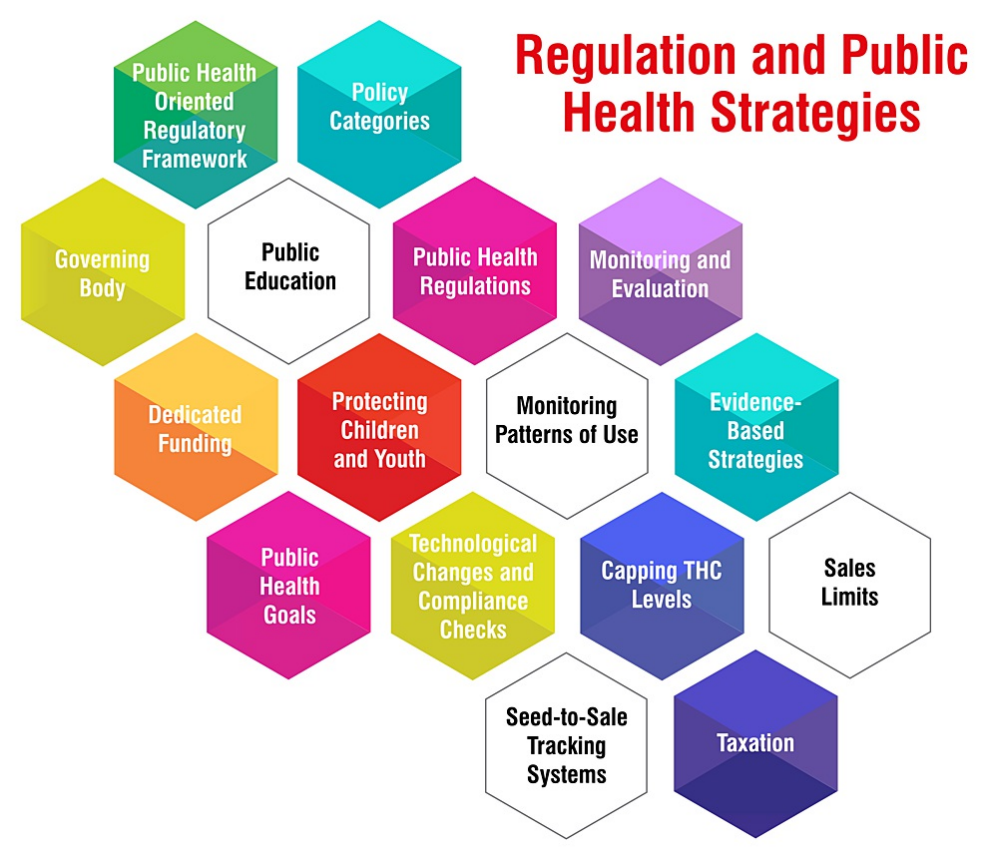
Regulation and public health strategies This figure is self-created by the corresponding authors.

Regulating cannabis from a public health perspective entails drawing lessons from the regulation of alcohol and tobacco to safeguard public health while balancing potential benefits and harms within a non-prohibition-based regulatory framework [35,36]. Insights from alcohol and tobacco control advocate strategies such as limiting sales outlets, imposing restrictions on business hours, and ensuring well-trained staff to mitigate sales to minors [35]. Establishing a governing body explicitly oriented toward public health objectives, akin to a "Cannabis Control Commission," can help ensure that regulations align with public health goals [35]. To protect public health, regulations should prioritize monitoring and surveillance to comprehend the effects of policy changes on health outcomes. This encompasses evaluating the potential adverse health impacts of cannabis, assessing the repercussions of policy alterations, and informing the development of public health initiatives and policies [37]. Public health regulations should focus on shielding children, adolescents, and vulnerable populations by implementing constraints that curtail cannabis use autonomy and prevent harm from passive exposure to cannabis smoke [37]. Furthermore, some revenue generated through cannabis regulation should be dedicated to health and social initiatives like early childhood education and mental health services [37]. In crafting cannabis legislation, policymakers should contemplate measures such as restricting high-potency cannabis use, enforcing sales limits, levying taxes based on potency, and implementing seed-to-sale tracking systems to promote responsible use and safeguard public health [38]. It is imperative for states or jurisdictions legalizing cannabis to do so within a robust public health-centered regulatory framework that safeguards against harms associated with legalization, ensuring that cannabis products are not accessible to individuals under the age of 21 [39]. By embracing evidence-based strategies and regulations rooted in public health principles, jurisdictions can navigate the intricacies of cannabis regulation while upholding the well-being of their populations. Regulation and public health strategies are shown in Figure 1.

Legal issues and challenges

Critique of Current Laws Regarding Marijuana Possession, Cultivation, and Distribution

The current legal framework surrounding marijuana possession, cultivation, and distribution in India presents a multifaceted landscape characterized by divergent viewpoints on legalization [21,40]. In recent times, there has been a surge in discussions and movements advocating for the legalization of cannabis, citing its potential for economic growth, job creation, and medicinal benefits as compelling reasons to reconsider existing laws [20]. Critics of the current laws underscore the challenges associated with regulating illegal cultivation, production, sale, quality control, and marketing that would accompany legalization. The necessity for stringent regulations, including age restrictions for procurement and controlling the THC content in cannabis products, presents significant obstacles [21]. Concerns regarding potential misuse, addiction, public health ramifications, and the capacity to regulate within the existing criminal justice and healthcare systems have also been raised [40]. Moreover, the debate contemplates the insights gleaned from other nations that have legalized recreational cannabis use and whether India is adequately equipped for such a transition [21]. The ongoing discourse reflects evolving societal attitudes and underscores the necessity for a balanced approach to cannabis regulation in India.

Human Rights Implications and Disproportionate Enforcement of Marijuana Laws

The human rights implications and disproportionate enforcement of marijuana laws in India present complex and multifaceted issues. The legal status of cannabis in the country is intricate, influenced by a combination of historical context and evolving perspectives [20,41]. Despite existing regulations, there are notable loopholes; for example, bhang, an extract of the cannabis plant, remains legal and holds cultural significance in India [20,21]. However, concerns persist regarding potential misuse and public health implications [42]. Criticism has been directed toward the legal framework and enforcement of marijuana laws in India, particularly for their disproportionate impact on certain communities. This has sparked calls for reform and more equitable enforcement practices [41]. The human rights implications of marijuana laws in India are substantial. Viewing these laws through a human rights lens can help address a fundamental tension between the state and the individual, focusing not only on the legality of cannabis but also on broader concerns [41]. International comparisons suggest that adopting a human rights perspective can lead to a more comprehensive approach to medicinal cannabis use, promoting policies that are oriented toward public health [41].

International Legal Obligations and Conventions

International law comprises a complex system of rules and principles governing the relations between sovereign states, international organizations, and individuals across various domains, including trade, human rights, diplomacy, environmental preservation, and war crimes [43]. Treaties are foundational elements of international law, constituting binding agreements between nations that delineate rights and obligations [43]. The Vienna Convention on the Law of Treaties codifies the rules governing treaties, emphasizing good faith interpretation and adherence to the drafters' intentions [43]. To become parties to treaties, states must ratify them, demonstrating their approval and commitment to abide by the provisions outlined in the treaties [43]. International law operates based on consent, with treaties as vital instruments for fostering cooperation on defense, environmental conservation, and trade [44]. Customary law, general principles of law, and judicial decisions also contribute to the sources of international law alongside treaties, helping establish norms and guidelines for states to follow in their interactions. The enforcement of international law lacks a centralized judicial system for compulsory enforcement. Nevertheless, institutions, such as the International Court of Justice, provide a platform for peaceful dispute resolution among member states by international law [45]. States must accept the jurisdiction of such bodies to be bound by their decisions. Compliance with treaties hinges on states' consent to be bound by the terms outlined in the treaty, as expressed through specific actions delineated within the treaty itself [45].

Public opinion and policy perspectives

Surveys and Studies on Public Attitudes Toward Marijuana Legalization

Various factors influence support and opposition to cannabis legalization in the United States. According to a study conducted in Michigan, approximately 48.1% of respondents supported cannabis legalization. Supporters often cited perceptions of marijuana as being less harmful than other substances, the need for criminal justice reform, and the potential for generating tax revenue. Conversely, opponents of legalization often highlighted the harms associated with marijuana use [46]. The demographic impact on public opinion regarding marijuana legalization is significant, with variations observed across age groups, political affiliations, and racial or ethnic backgrounds [47,48]. Public support for cannabis legalization has experienced a notable shift over the years, with a significant increase observed since the 1990s. By 2014, support for legalization had reached 57.4%, indicating a steady upward trend. Factors contributing to this growth include changing perceptions of marijuana's medical benefits and a broader societal shift in attitude toward legalization [48]. Moreover, there is considerable support for easing penalties related to marijuana offenses among Americans. A survey revealed that a majority of adults favor the release of individuals detained for marijuana-related offenses and support the removal or expungement of such offenses from criminal records [47]. However, attitudes toward marijuana legalization also reflect a generational divide, with differing perspectives observed among different age groups [48].

Political Discourse and Challenges in Policy Reform

Political discourse and the challenges in policy reform are multifaceted and influenced by various factors. Public discourse plays a pivotal role in shaping perspectives and decisions regarding policy. Challenges in policy research encompass issues such as the erosion of public trust in government, devolution without thorough evaluation, and the emergence of morality-driven politics [49]. However, the public also recognizes strengths within the political system, including principles of democracy, freedom of speech, and checks and balances designed to regulate the exercise of power [50]. To address these challenges, longitudinal studies are proposed to provide insights into complex social issues and offer evidence-based solutions for effective policy formulation and implementation [49]. Political discourse is intrinsically linked to public opinion, the obstacles encountered in policy research, and perceptions of the political system. Mitigating these challenges necessitates a commitment to transparency, accountability, evidence-based policymaking, and a focus on long-term strategies for addressing complex social issues [49].

Perspectives From Various Stakeholders: Medical Professionals, Law Enforcement, and Policymakers

Interviews conducted with law enforcement officers in states like Colorado and Oregon have shed light on significant concerns regarding the increasing potency of marijuana products and the strain placed on criminal justice resources due to the demands associated with other drugs, such as heroin and methamphetamine [51]. The evolving legal status of marijuana has underscored the necessity for comprehensive data to assess these impacts across different agencies and over extended periods [51]. A decentralized data collection approach is imperative to grasp these intricate issues fully, recognizing the resource variations and barriers encountered across states [51]. Public opinion and policies regarding medical cannabis are notably influenced by beliefs concerning its medical effects. The perception of cannabis as having therapeutic benefits is closely linked with support for the legalization of medical cannabis [52]. Recent evidence affirming the medicinal advantages of cannabis may further bolster public confidence in its therapeutic properties and bolster support for legalization efforts [52]. Various stakeholders, including law enforcement officials, policymakers, and medical professionals, hold diverse perspectives regarding marijuana legalization. Medical professionals, in particular, stress the significance of beliefs regarding the medical benefits of cannabis in shaping public support for policies related to medical cannabis [52].

Case studies and comparative analysis

Review of States or Countries Where Marijuana Has Been Legalized or Decriminalized

Information on countries and states worldwide regarding the legalization and decriminalization of marijuana is described in Table 1.

**Table 1:** The information on countries and states worldwide regarding the legalization and decriminalization of marijuana

Country/State	Legalization Status	Details
Canada	Legalized	They legalized all forms of cannabis at the federal level in 2018. Allows for both medicinal and recreational use [53].
Uruguay	Legalized	Legalized recreational cannabis in December 2013. Licensed sales started in July 2017 [54].
Thailand	Legalized (Medical)	Legalized marijuana for medical use in 2018, with some regulations still in place [55].
Argentina	Legalized (Medical)	Legalized medical marijuana in 2017 and decriminalized small amounts for personal use in 2022 [56].
Netherlands	Tolerated (Recreational)	While technically illegal, the Netherlands has tolerated the sale and consumption of cannabis since the 1970s, particularly in Amsterdam's coffee shops [57].
Germany	Partially Legalized (Medical)	Allows medical use under tight regulations but prohibits recreational use [58].
Italy	Legalized (Medical)	Legalized marijuana for medicinal purposes only since 2013 [59].
Belgium	Partially Legalized	Allows the cultivation of one female plant since 2003 [60].
United States	Varies (Recreational)	Several states, such as Colorado, Washington, California, and others, have approved legal recreational marijuana use [61].
	Varies (Medical)	Thirty-eight states, four territories, and Washington, DC, have legalized medical marijuana use [62].
Mexico	Decriminalized	Decriminalized possession of small amounts of marijuana and moving toward full legalization [63].

Cultural and Societal Adjustments After Legalization

Cultural differences: Cultural factors significantly shape the manifestation of Cannabis Use Disorders (CUDs), with diverse legal and social climates influencing individuals' behaviors toward seeking treatment [64]. For instance, Australia's harm-reduction policy and public healthcare system may have a distinct impact on treatment outcomes compared to the United States [64]. These differences underscore the importance of considering cultural contexts in addressing CUDs and tailoring interventions to meet the unique needs of various communities.

Perception of risks: The perception of the risks associated with cannabis use can vary across cultures, leading to differing attitudes and behaviors regarding its consumption [64]. For example, there has been a decrease in the perceived risk of cannabis use among high-school students in the United States over the past two decades, prompting discussions about the effectiveness of criminalization as a deterrent [64]. Understanding these cultural variations in risk perception is essential for developing effective prevention and intervention strategies.

Modernization and secularization: In India, significant social and cultural shifts driven by modernization and secularization reshape traditional values and practices, influencing various aspects such as language use, religious affiliations, and societal structures [65]. These changes have profound implications for national identity formation and societal dynamics, impacting attitudes toward cannabis use and regulation. It is essential to recognize these evolving cultural dynamics when addressing issues related to cannabis legalization and public health policies.

Legal implications: The potential legalization of recreational cannabis in India raises complex legal implications, particularly concerning the readiness of existing criminal justice and healthcare systems to regulate its use [21] effectively. Policy debates surrounding legalization emphasize the importance of establishing clear regulations, addressing public health considerations, and implementing stringent laws to mitigate potential risks associated with cannabis use [21]. Ensuring adequate infrastructure and resources are in place is crucial for navigating the legal and societal challenges that may arise from cannabis legalization.

## Conclusions

In conclusion, this comprehensive review underscores the profound historical and cultural significance of marijuana in India, juxtaposed against the restrictive legal framework that currently governs its use. From ancient rituals to modern medicinal applications, marijuana has been deeply intertwined with Indian society for millennia. However, the current legal status under the NDPS Act places stringent restrictions on its possession, cultivation, and distribution, resulting in adverse social consequences and hindrances to medical research. The evidence presented throughout this review highlights the need for policymakers and legislators to reevaluate and reform existing laws, considering the potential benefits of legalization and regulation. By adopting evidence-based policies and prioritizing public health, India can harness the therapeutic potential of marijuana, promote social equity, and stimulate economic growth. Furthermore, a vision for the future of marijuana regulation in India should encompass harm reduction, social justice, and innovation principles, positioning the country as a leader in cannabis research and sustainable cultivation practices. With bold and visionary leadership, India can navigate the complexities of marijuana regulation, unlocking its full potential as a “miracle crop” for the benefit of its people and society as a whole.

## References

[1] (2024). The ganja culture of India. https://www.cannabisculture.com/content/2019/12/09/the-ganja-culture-of-india/.

[2] Bania G (2022). Shifts in therapeutic practices and decline of medicinal cannabis in Indian North-Eastern Frontier (1826-1925). J Cannabis Res.

[3] Nayak P, Pantvaidya G, Ranganathan P, Jiwnani S, Joshi S, Gogtay NJ (2023). Clinical studies with Cannabis in India - A need for guidelines for the investigators and ethics committees. Perspect Clin Res.

[4] (2024). Punishment for offences. https://www.dor.gov.in/narcoticdrugspsychotropic/punishment-offences.

[5] Ledger E (2024). Cannabis use in the ancient world: Ancient India. https://canex.co.uk/cannabis-use-in-the-ancient-world-ancient-india/.

[6] (2024). Shiva and the cannabis. https://timesofindia.indiatimes.com/readersblog/mamta-rana/shiva-and-the-cannabis-6941/.

[7] Karki P, Rangaswamy M (2023). A review of historical context and current research on cannabis use in India. Indian J Psychol Med.

[8] (2024). The history and evolution of hemp cultivation. https://indiahemporganics.com/blogs/general-blog/the-history-and-evolution-of-hemp-cultivation.

[9] (2024). History of cannabis. https://www.sydney.edu.au/lambert/medicinal-cannabis/history-of-cannabis.html.

[10] Lamp J (2024). Cannabis in religious practice [Guide for beginners]. Union Square Lamp Co.

[11] Burdette AM, Webb NS, Hill TD, Haynes SH, Ford JA (2018). Religious involvement and marijuana use for medical and recreational purposes. J Drug Issues.

[12] Yeterian JD, Bursik K, Kelly JF (2018). "God put weed here for us to smoke": a mixed-methods study of religion and spirituality among adolescents with cannabis use disorders. Subst Abus.

[13] Chattopadhyaya U (2022). Reading cannabis in the colony: law, nomenclature, and proverbial knowledge in British India. The Social History of Alcohol and Drugs.

[14] (2024). A detailed overview of narcotic drugs and psychotropic substances act,1985. https://timesofindia.indiatimes.com/readersblog/legalangle/a-detailed-overview-of-narcotic-drugs-and-psychotropic-substances-act1985-45878/.

[15] (2024). Narcotic drugs and psychotropic substances (NDPS) Act, 1985. https://testbook.com/ias-preparation/ndps-act.

[16] (2024). Narcotic drugs and psychotropic substances act, 1985. https://www.ojp.gov/ncjrs/virtual-library/abstracts/narcotic-drugs-and-psychotropic-substances-act-1985-commentary.

[17] Ambekar A, Gautam M, Matcheswalla Y, Kar S, Kadam K (2022). Medicolegal issues with reference to NDPS and MHCA in management and rehabilitation of persons with substance use disorders. Indian J Psychiatry.

[18] (2024). NDPS - classification of drugs. https://bhattandjoshiassociates.com/ndps-classification-of-drugs/.

[19] (2024). Offences and penalties under NDPS Act, 1985. https://legalserviceindia.com/legal/article-10956-offences-and-penalties-under-ndps-act-1985.html.

[20] Sarma CLG-K, Sarma R, Gupta S (2023). A general introduction to cannabis law in India. Lexology.

[21] Chithra NK, Bojappen N, Vajawat B (2023). Legalization of recreational cannabis: is India ready for it?. Indian J Soc Psychiatry.

[22] (2024). Where does India stand legally on medical cannabis. https://hempstreet.in/blog/where-does-india-stand-legally-on-medical-cannabis/.

[23] Hamid Z (2024). Cannabis in India: Does the law need to catch up with reality. https://www.thehindu.com/podcast/cannabis-in-india-does-the-law-need-to-catch-up-with-reality/article67388456.ece.

[24] Project MP (2024). Marijuana prohibition facts. https://www.mpp.org/issues/legalization/marijuana-prohibition-facts/.

[25] (2024). The effect of state marijuana legalizations. https://www.cato.org/policy-analysis/effect-state-marijuana-legalizations-2021-update.

[26] 20 20 (2024). The case for legalizing medical marijuana in India: Health, economic, and socio-political perspectives. https://www.linkedin.com/pulse/case-legalizing-medical-marijuana-india-health-economic-sunin-sunny/.

[27] Vasudeva V (2024). Apple country Himachal Pradesh gets ground ready for cannabis cultivation. https://www.thehindu.com/news/national/other-states/himachal-pradesh-inches-closer-to-cannabis-cultivation/article67291491.ece.

[28] Farrelly KN, Wardell JD, Marsden E, Scarfe ML, Najdzionek P, Turna J, MacKillop J (2023). The impact of recreational cannabis legalization on cannabis use and associated outcomes: a systematic review. Subst Abuse.

[29] (2024). What are the health benefits and risks of cannabis. https://www.medicalnewstoday.com/articles/320984.

[30] MD PG (2024). Medical marijuana. https://www.health.harvard.edu/blog/medical-marijuana-2018011513085.

[31] (2024). 7 potential health benefits of cannabis. https://www.jwu.edu/news/2021/09/7-potential-health-benefits-of-cannabis.html.

[32] Sewell RA, Poling J, Sofuoglu M (2009). The effect of cannabis compared with alcohol on driving. Am J Addict.

[33] (2024). Addiction. https://www.cdc.gov/marijuana/health-effects/addiction.html.

[34] Canada H (2024). Cannabis health effects. https://www.canada.ca/en/services/health/campaigns/cannabis/health-effects.html.

[35] Haden M, Emerson B (2014). A vision for cannabis regulation: a public health approach based on lessons learned from the regulation of alcohol and tobacco. Open Med.

[36] Pacula RL, Kilmer B, Wagenaar AC, Chaloupka FJ, Caulkins JP (2014). Developing public health regulations for marijuana: lessons from alcohol and tobacco. Am J Public Health.

[37] (2024). A public health approach to regulating commercially legalized cannabis. https://www.apha.org/policies-and-advocacy/public-health-policy-statements/policy-database/2021/01/13/a-public-health-approach-to-regulating-commercially-legalized-cannabis.

[38] Rosalie Liccardo Pacula P, Seema (Choksy) Pessar MPP, Zhu J, Rosanna Smart P (2022). Federal regulations of cannabis for public health in the United States. Published Online First: 18 July.

[39] (2024). Preventing marijuana use among youth. https://www.samhsa.gov/resource/ebp/preventing-marijuana-use-among-youth.

[40] Sarkar V (2024). A one-stop homegrown guide to marijuana laws in India. https://homegrown.co.in/homegrown-voices/a-one-stop-homegrown-guide-to-marijuana-laws-in-india.

[41] Bone M, Seddon T (2016). Human rights, public health and medicinal cannabis use. Crit Public Health.

[42] (2024). Is weed or marijuana legal in India. https://timesofindia.indiatimes.com/readersblog/lawpedia/is-weed-or-marijuana-legal-in-india-50397/.

[43] (2024). International law. LII/ legal information institute. https://www.law.cornell.edu/wex/international_law.

[44] (2024). International law. https://www.britannica.com/topic/international-law.

[45] Nations U (2024). Uphold international law. https://www.un.org/en/our-work/uphold-international-law.

[46] Resko S, Ellis J, Early TJ, Szechy KA, Rodriguez B, Agius E (2019). Understanding public attitudes toward cannabis legalization: qualitative findings from a statewide survey. Subst Use Misuse.

[47] Schaeffer K (2024). 7 facts about Americans and marijuana. https://www.pewresearch.org/short-reads/2023/04/13/facts-about-marijuana/.

[48] Green TV (2024). Americans overwhelmingly say marijuana should be legal for medical or recreational use. https://www.pewresearch.org/us-politics-marijuana-newyork/.

[49] (2024). Common policy problems and what researchers can do about them. https://eprints.lse.ac.uk/107179/1/impactofsocialsciences_2020_10_29_common_policy_challenges_and_what.pdf.

[50] Center PR: 1 (2024). The biggest problems and greatest strengths of the U.S. political system. Politics & Policy.

[51] Stanton DL, Makin D, Stohr M (2022). Law enforcement perceptions of cannabis legalization effects on policing: Challenges of major policy change implementation at the street level. Contemp Drug Probl.

[52] Sznitman SR, Bretteville-Jensen AL (2015). Public opinion and medical cannabis policies: examining the role of underlying beliefs and national medical cannabis policies. Harm Reduct J.

[53] Canada H (2024). Taking stock of progress: Cannabis legalization and regulation in Canada. https://www.canada.ca/en/health-canada/programs/engaging-cannabis-legalization-regulation-canada-taking-stock-progress/document.html.

[54] Laqueur H, Rivera-Aguirre A, Shev A (2020). The impact of cannabis legalization in Uruguay on adolescent cannabis use. Int J Drug Policy.

[55] Thepgumpanat P, Wongcha-um P (2024). Thailand to ban recreational cannabis use by year-end, health minister says. https://www.reuters.com/world/asia-pacific/thailand-ban-recreational-cannabis-use-by-year-end-says-health-minister-2024-02-29/.

[56] Aguilar Ó, Díaz MC, Romero L (2022). Citizen science towards the regulation of medical cannabis in Argentina. Tapuya: Lat Am Sci Technol Soc.

[57] Visram T (2024). The Netherlands was once a cannabis pioneer, but it still hasn’t legalized weed. https://www.fastcompany.com/90832031/the-netherlands-was-once-a-cannabis-pioneer-but-it-still-hasnt-legalized-weed-what-happened.

[58] (2024). German lawmakers appove new law to partially legalise cannabis. https://www.euronews.com/health/2024/02/23/german-lawmakers-to-debate-and-vote-on-legalising-cannabis.

[59] July 2017 BM (2024). Cannabis legalization world map. https://www.cannabisbusinesstimes.com/article/cannabis-legalization-world-map/.

[60] (2024). Cannabis law and legislation in Belgium. https://cms.law/en/int/expert-guides/cms-expert-guide-to-a-legal-roadmap-to-cannabis/belgium.

[61] Pacula RL, Smart R (2017). Medical marijuana and marijuana legalization. Annu Rev Clin Psychol.

[62] (2024). State medical cannabis laws. https://www.ncsl.org/health/state-medical-cannabis-laws.

[63] (2009). Mexico decriminalizes small-scale drug possession. HIV AIDS Policy Law Rev.

[64] Prashad S, Milligan AL, Cousijn J, Filbey FM (2017). Cross-cultural effects of cannabis use disorder: evidence to support a cultural neuroscience approach. Curr Addict Rep.

[65] (2024). Major social & cultural trends in India - Lesson. https://study.com/academy/lesson/major-social-cultural-trends-in-india.html.

